# Structural and thermodynamic analyses of a novel β‐1,2‐glucan binding mode in the ABC transporter solute‐binding protein Chy400_4166 from *Chloroflexus aurantiacus*

**DOI:** 10.1111/febs.70576

**Published:** 2026-05-10

**Authors:** Kazuya Kato, Tatsuya Kaneko, Rintaro Hirayama, Nobukiyo Tanaka, Hiroyuki Nakai, Hidetaka Torigoe, Masahiro Nakajima

**Affiliations:** ^1^ Faculty of Science and Technology, Department of Applied Biological Science Tokyo University of Science Noda Chiba Japan; ^2^ Faculty of Agriculture Niigata University Japan; ^3^ Faculty of Science, Department of Applied Chemistry Tokyo University of Science Japan

**Keywords:** ABC transporter, cyclic β‐1,2‐glucan, isothermal titration calorimetry, solute‐binding protein, β‐1,2‐glucooligosaccharide

## Abstract

β‐1,2‐Glucans are glucose polymers widely distributed in nature and play various physiological roles in the interactions between organisms such as pathogenicity and symbiosis. While various β‐1,2‐glucan‐degrading enzymes have been identified recently, transporters incorporating β‐1,2‐glucans are still poorly characterized. In this study, we have found a β‐1,2‐glucan binding protein of ABC transporter from *Chloroflexus aurantiacus* Y‐400‐fl, a filamentous anoxygenic phototrophic bacterium. The protein showed a clear affinity for linear β‐1,2‐glucan in the gel shift assay. Isothermal titration calorimetric analysis revealed high binding affinities for both linear and cyclic β‐1,2‐glucans, unlike for the barley β‐glucan. The recorded binding constants were high for the binding of the ABC transporter to β‐1,2‐glucans. The observed unfavorable negative entropy change may have resulted from conformational restraints upon complex formation. Complex structures with linear β‐1,2‐glucan and cyclic β‐1,2‐glucans with degrees of polymerization of 17–20 were obtained using X‐ray crystallography. Ten glucose units, designated A–J from the nonreducing end, were shared among the substrates in the complexes. Unit G is recognized by W74, W308, and D336, which are highly conserved residues within the phylogenetic group Chy400_4166. The substrate‐binding mode of Chy400_4166 is completely different from that of the β‐1,2‐glucooligosaccharide‐binding protein from *Listeria innocua*. The discovery of a new type of β‐1,2‐glucan‐related binding protein has expanded our understanding of the metabolism of β‐1,2‐glucans.

## Graphical abstract

Cyclic β‐1,2‐glucan (CβG)‐associated solute‐binding protein of type I ABC transporter was originally identified in *Chloroflexus aurantiacus*, a phototrophic bacterium. Crystal structures in complex with CβGs revealed unique binding modes that are fundamentally distinct from that of the short β‐1,2‐glucooligosaccharides‐binding protein. These complexes highlight the structural features that favor cyclic over linear forms while accommodating CβGs of various chain lengths.

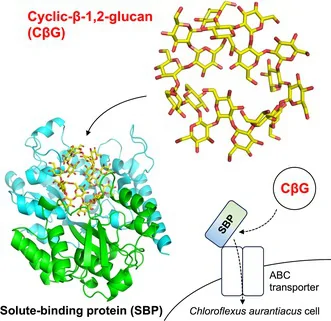

**Abbreviations**
CM: carboxymethyl
CβG: cyclic β‐1,2‐glucan
DP: degree of polymerization
GH: glycoside hydrolase
ITC: isothermal titration calorimetry
LiSO‐BP: SO‐BP from *Listeria innocua*
LβG: linear β‐1,2‐glucan
LβG121: LβGs with average DP 121
OPG: osmoregulated periplasmic glucans
RMSD: root mean square deviation
SBP: solute‐binding protein
SGL: endo β‐1,2‐glucanase
SO‐BP: Sop_n_s‐binding protein
SOGP: 1,2‐β‐oligoglucan phosphorylase
Sop_n_
: β‐1,2‐glucooligosaccharide
TMD: transmembrane domain

## Introduction

β‐1,2‐Glucans, which are glucose polymers found mainly in cyclic forms, are widely distributed in nature [1]. Although β‐1,2‐glucans are less abundant in nature than cellulose and β‐1,3‐glucans, β‐1,2‐glucans play important physiological roles in processes ranging from symbiosis and pathogenicity to osmoregulation [2]. For example, *Brucella abortus* uses cyclic β‐1,2‐glucans (CβGs) to prevent a *Brucella*‐containing vacuole from fusion with a lysosome [3]. This interference confers resistance to the host defense system against bacteria. Cyclic glucans produced by *Xanthomonas* species, phytopathogens, which feature a single β‐1,2‐glucosidic linkage substituted with an α‐1,6‐linked glucose unit, are known as CβGαs [4, 5]. *Xanthomona*s *campestris* produces CβGα with a degree of polymerization (DP) of 16. The bacterial mutant defective in the production of CβGα with DP16 (CβG16α; hereafter, DPs are indicated immediately following the name) loses pathogenicity toward model plants, such as *Nicotiana benthamiana* and *Arabidopsis thaliana* [6]. External supplementation of CβG16α to the mutant strain restores its pathogenicity. In *Escherichia coli*, short linear β‐1,2‐glucans (LβGs) with β‐1,6‐linked glucose side chains are called osmoregulated periplasmic glucans (OPG) [7]. The deletion of a gene presumably involved in OPG synthesis was shown to enhance the pathogenicity to silkworms and their resistance to antibiotics [8]. OPG are required for the phytopathogenicity of *Dickeya dadantii* (formerly *Erwinia chrysanthemi*) [9]. Recently, β‐1,2‐glucotrisaccharide was found to induce pattern‐triggered immunity in Arabidopsis, maize, and wheat [10].

Regarding β‐1,2‐glucan‐associated enzymes, past research on CβG synthases is well‐established, whereas the exploration of degrading enzymes has remained limited [11, 12, 13]. A new glycoside phosphorylase acting on LβGs (1,2‐β‐oligoglucan phosphorylase, SOGP, see Data S1: supplementary note for abbreviation) was found from *Listeria innocua*, a Gram‐positive bacterium [14]. Subsequently, a large‐scale production method of LβGs was developed using this enzyme [15, 16]. Furthermore, endo β‐1,2‐glucanases (SGLs) hydrolyzing β‐1,2‐glucans to release β‐1,2‐glucooligosaccharides (Sop_n_s, see Data S1: supplementary note for abbreviation) were identified from bacterial and fungal sources by culturing the organisms with the produced β‐1,2‐glucans as a sole carbon source [17, 18]. Because these enzymes possess phylogenetically distinct amino acid sequences, they have been assigned to new glycoside hydrolase (GH) families GH144 and GH162 for bacterial and fungal SGLs, respectively [19, 20]. Recently, the identification of four additional distantly related GH families (GH189, GH192, GH193, and GH194) has significantly expanded the known diversity of GH enzymes [11, 21]. Another phylogenetically novel SGL was identified in *E. coli*, which led to the establishment of a new GH family (GH186) [22]. In addition, CβG16α synthase from *X. campestris* was found in the GH186 family [1, 23]. These findings on SOGP and the new GH families facilitated the investigation of homologs within these families and gene clusters. Consequently, various new β‐1,2‐glucan‐associated enzymes were found, and their structure–function relationships were elucidated [19, 24, 25]. For example, a sophorose (β‐1,2‐glucodisaccharide)‐releasing enzyme and a transglycosylase transferring a glucose unit between β‐1,2‐glucooligosaccharides have been found in GH144 and GH35, respectively [26, 27].

In contrast to degrading enzymes, β‐1,2‐glucan‐associated binding proteins such as transporters have been poorly investigated. ABC transporters are one of the primary bacterial transporter systems. They typically feature two transmembrane domains (TMD) that form pathways for substrate translocation. Based on their TMD sequences, ABC transporters are classified into seven types (I to VII) [28]. Types I–III function as substrate importers, whereas types IV and V function predominantly as exporters. Types VI and VII have been identified as extractors and mechanotransmitters, respectively. In the type I and II systems, the importers operate in conjunction with solute‐binding proteins (SBPs), which capture and deliver specific substrates to TMDs for translocation. In Gram‐negative bacteria, SBPs are not always captured by TMDs. Although the affinity for substrates varies significantly among SBPs, type I SBPs typically exhibit a lower affinity for substrates than type II SBPs. This suggests that type I ABC transporters are primarily responsible for the uptake of the relatively abundant compounds. Ultimately, the functional properties of SBP determine the substrate specificities of both type I and type II ABC transporters.

To date, only two previous studies have been conducted on ABC transporters associated with β‐1,2‐glucans. The gene cluster of *sogp* (*lin1839*) gene from *L. innocua* includes a set of genes encoding a putative type I ABC transporter (*lin1841*–*1843*) [14, 29]. Lin1841, an SBP homolog, was identified as a Sop_n_s‐binding protein (SO‐BP) and is hereafter referred to as LiSO‐BP [30]. Complemented by the biochemical characterization of Lin1840, a β‐glucosidase with preference for Sop_n_s, and SOGP (Lin1839), this gene cluster is proposed to be responsible for Sop_n_s metabolism. Another example is the type IV exporter in *Brucella abortus* [31]. It functions by transporting CβGs produced in the periplasm to outside of the cell.

In addition to the aforementioned examples, *C. aurantiacus* Y‐400‐fl [32], a filamentous anoxygenic phototrophic bacterium, harbors a different type of potential β‐1,2‐glucan‐associated SBP based on the phylogenetic classification. The bacterium has two *sgl* genes (*chy400_4174* and *chy400_4176*) belonging to GH144; the former has been reported to be an SGL [15]. The gene cluster of the two *sgl* genes contains two sets of genes encoding putative type I ABC transporters (Fig. S1). Among these transporters, *chy400_4166* and *chy400_4167* are genes encoding putative solute‐binding proteins. However, most substrate recognition residues in Lin1841 are not conserved in Chy400_4166 or Chy400_4167, thus suggesting that these proteins have different properties from that of Lin1841. In this study, we have found that Chy400_4166 is a CβG‐binding protein biochemically and solved complex structures with CβGs to understand structure–function relationships.

## Results

### Substrate specificity of Chy400_4166

In order to investigate the substrate specificity of Chy400_4166 and Chy400_4167, a gel shift assay was performed against the two proteins by Native‐PAGE using gels containing soluble polysaccharides (Fig. 1). Chy400_4167 did not show band shifts between the gels with and without the polysaccharides tested (data not shown). Contrarily in Chy400_4166, LβGs with DP 121 (LβG121, see Materials and Methods for details) shifted the band upward remarkably. Laminarin, barley β‐glucan and lichenan also shifted the bands upward to some extent. CβGs did not work for shifting a band probably due to small DPs. It is because the extent of band shift depended on DPs of LβGs. Thus, to understand the binding affinity in detail, thermodynamic analysis using isothermal titration calorimetry (ITC) is required.

**Fig. 1:**
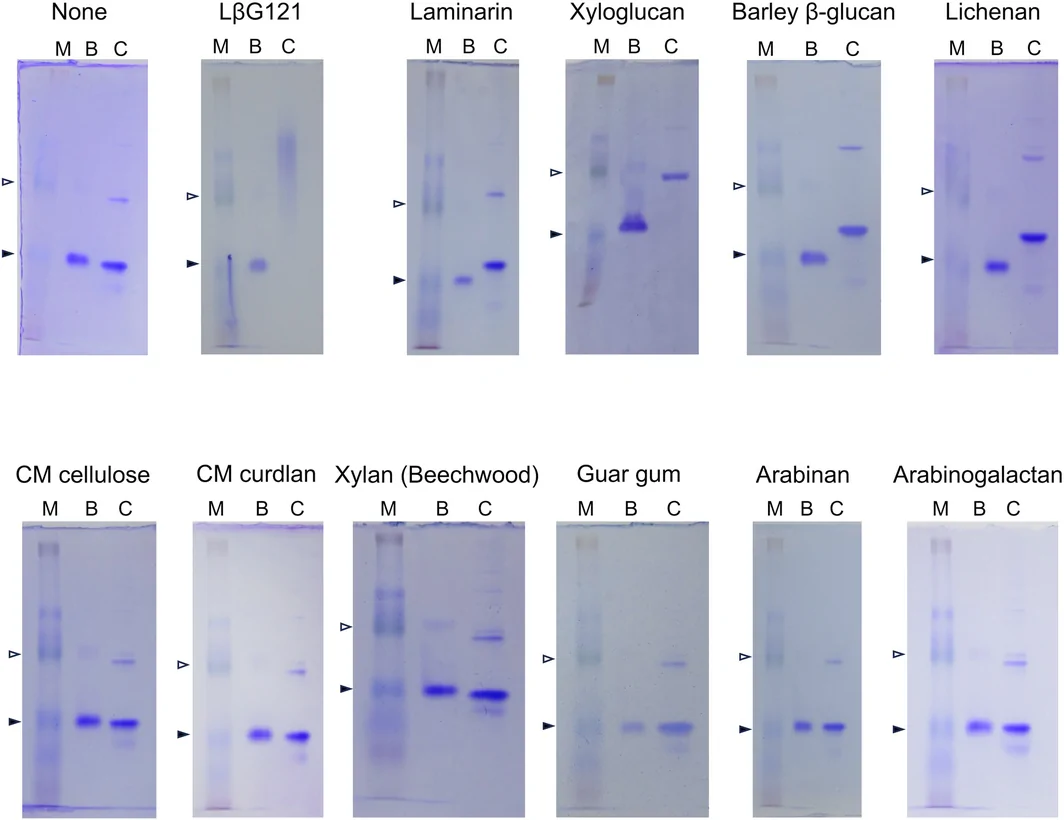
Gel shift assay of Chy400_4166 toward polysaccharides. Lanes M, B, and C show the marker proteins (left), bovine serum albumin (middle), and Chy400_4166 (right), respectively. Arrowheads indicate phosphorylase‐b (white, 100 kDa) and bovine serum albumin (black, 71 kDa) as the prestained marker proteins. The gel images are representative of independent experiments with similar results.

### Thermodynamic analyses for the direct binding of Chy400_4166 to a series of β‐glucans by ITC

To explore the molecular mechanism underlying the binding of Chy400_4166 to a series of β‐glucans in more detail, we investigated the direct binding of Chy400_4166 to a series of β‐glucans via ITC. We examined the thermodynamic properties for the binding of Chy400_4166 to a series of β‐glucans in 20 mM sodium phosphate buffer (pH 7.0) at 25 °C via ITC (Fig. 2). Fig. 2A,C,E,G,I,K shows the typical ITC profile for the titration of Chy400_4166 into (A) CβG17, (C) CβG18, (E) CβG19, (G) CβG20, (I) LβG121, and (K) barley β‐glucan after the subtraction of the heat of dilution of Chy400_4166. The area under each peak in Fig. 2A,C,E,G,I,K was integrated, and the integrated values were then divided by the number of moles of Chy400_4166 solution. The resulting values were plotted as a function of the [Chy400_4166]/[β‐glucan] molar ratio to obtain Fig. 2B,D,F,H,J,L. The resulting titration plot was fitted to a sigmoidal curve using the nonlinear least‐squares method. The stoichiometry (*N*), binding constant (*K*_a_), and enthalpy change (∆*H*) values for the binding of Chy400_4166 to the series of β‐glucans were calculated from the fitted curve, as previously described [33]. The Gibbs free energy change (∆*G*) and entropy change (∆*S*) were calculated from the equation, ∆*G* = *–RT*ln*K*_a_ = ∆*H–T*∆*S*, where *R* is the gas constant and *T* is the temperature.

**Fig. 2:**
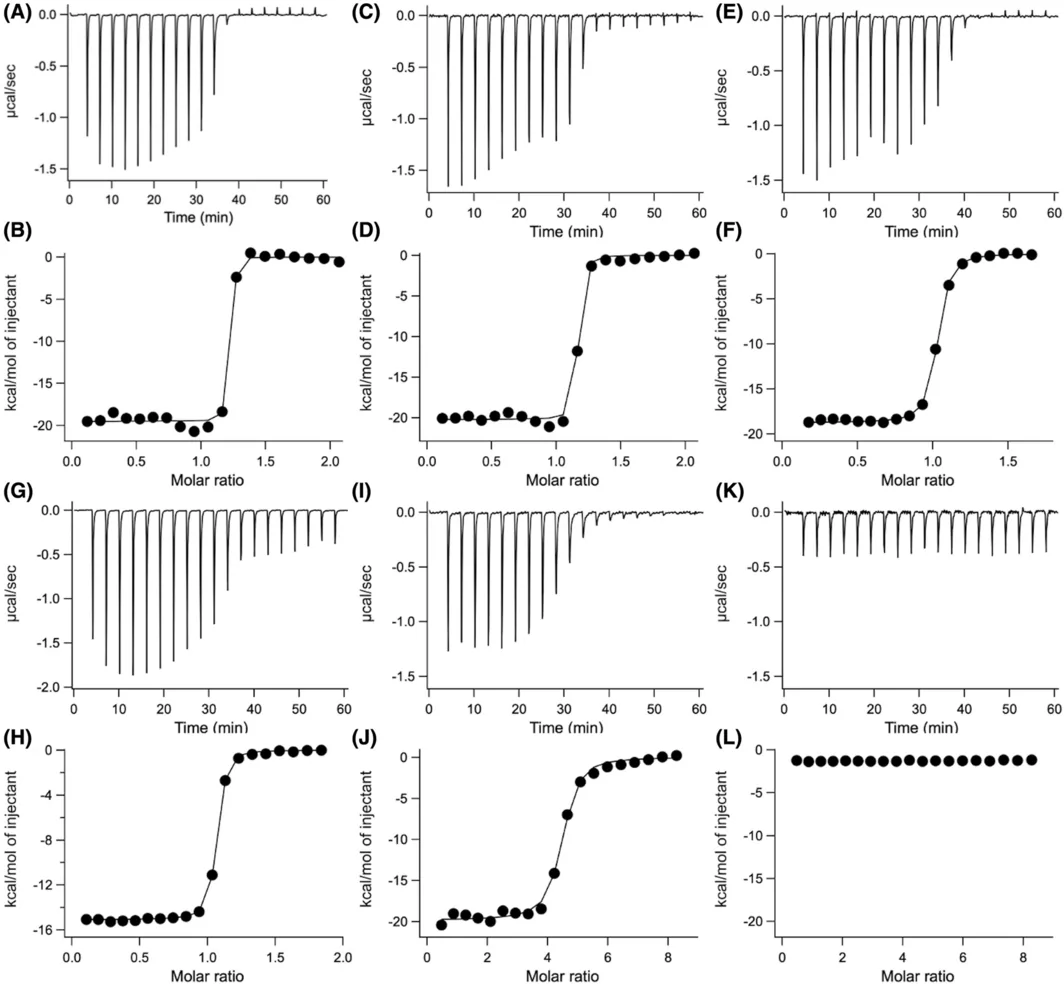
Thermodynamic analyses for the binding of Chy400_4166 to a series of β‐glucans at 25 °C and pH 7.0. (A, C, E, G, I, K) Typical isothermal titration calorimetry (ITC) profiles for the binding of Chy400_4166 to (A) CβG17, (C) CβG18, (E) CβG19, (G) CβG20, (I) LβG121, and (K) barley β‐glucan at 25 °C and pH 7.0 in 20 mm sodium phosphate buffer after the subtraction of the heat of dilution of Chy400_4166. Chy400_4166 solution [400 μm for (A), (C), (E), (G), (I) and 2400 μm for (K)] in 20 mm sodium phosphate buffer (pH 7.0) was injected 20 times in 2 μL increments into the β‐glucan solution [40 μm for (A), (C), (E), (G), 10 μm for (I), and 60 μm for (K)] in 20 mm sodium phosphate buffer (pH 7.0) over 4 s at 3 min intervals. The ITC profile for the injection of Chy400_4166 solution into the 20 mm sodium phosphate buffer (pH 7.0) was subtracted from those for the injection of Chy400_4166 solution into the β‐glucan solution to obtain (A), (C), (E), (G), (I), and (K). (B, D, F, H, J, L) Titration plots of (B), (D), (F), (H), (J), and (L) against the [Chy400_4166]/[β‐glucan] molar ratio obtained from the ITC profiles of (A), (C), (E), (G), (I), and (K), respectively. Data are fitted using the nonlinear least‐squares method. These are the representative ITC profiles obtained from the ITC instruments among three independent measurements. The obtained thermodynamic parameters are listed in Table 1 with average and standard deviation values.

The thermodynamic parameters at 25 °C for the binding of Chy400_4166 to the series of β‐1,2‐glucans obtained from Fig. 2B,D,F,H,J are summarized in Table 1. The obtained values of *N* for the CβGs (CβG17, CβG18, CβG19, and CβG20) were nearly 1, indicating that Chy400_4166 bound to the CβGs at a 1:1 molar ratio. The observed value of *N* for the LβG121 was around 4, showing that the number of Chy400_4166 bound to the LβG was significantly larger than that bound to the CβGs. On the other hand, the titration plot in Fig. 2L was not sigmoidal, in sharp contrast to the sigmoidal titration plots shown in Fig. 2B,D,F,H,J. The heat magnitude of the titration plot in Fig. 2L was nearly zero and was significantly smaller than those in Fig. 2B,D,F,H,J. These results have revealed that the binding ability of Chy400_4166 to barley β‐glucan was significantly smaller than that to the cyclic and linear β‐1,2‐glucans, although an upward shift in the bands was observed for barley β‐glucan in the gel shift assay (Fig. 1).

**Table 1:** Thermodynamic parameters for the binding of Chy400_4166 to a series of β‐1,2‐glucans at 25 °C and pH 7.0 in 20 mm sodium phosphate buffer, obtained from the ITC measurements^a^.

β‐1,2‐Glucan	*N*	*K*_a_ (M^−1^)	Δ*G* (kcal·mol^−1^)	Δ*H* (kcal·mol^−1^)	Δ*S* (cal·mol^−1^·K^−1^)
CβG17	1.17 ± 0.01	(4.15 ± 1.75) × 10^8^	−11.8 ± 0.32	−19.5 ± 0.18	−26.0 ± 1.7
CβG18	1.12 ± 0.01	(9.55 ± 2.88) × 10^7^	−10.9 ± 0.21	−20.2 ± 0.17	−31.2 ± 1.3
CβG19	1.04 ± 0.01	(1.37 ± 0.14) × 10^7^	−9.74 ± 0.06	−20.0 ± 0.19	−34.4 ± 0.9
CβG20	1.03 ± 0.01	(3.18 ± 0.33) × 10^7^	−10.2 ± 0.06	−15.1 ± 0.11	−16.3 ± 0.6
LβG121	4.31 ± 0.03	(6.19 ± 1.09) × 10^6^	−9.26 ± 0.11	−19.8 ± 0.21	−35.3 ± 1.1

^a^ These parameters are the average of three independent measurements.

The observed *K*_a_ values for the binding of Chy400_4166 to the cyclic and linear β‐1,2‐glucans (Table 1) were quite large for the binding of ABC transporter to β‐1,2‐glucans [30], supporting the specific binding of Chy400_4166 to the cyclic and linear β‐1,2‐glucans. Although both cyclic and linear β‐1,2‐glucans exhibited comparable activity in terms of whether they bind sufficiently, the *K*_a_ value for the binding to CβG17 was the largest among those to the cyclic and linear β‐1,2‐glucans (Table 1). When the number of DP for the CβG was increased, the *K*_a_ value for the binding to the CβG was decreased, although the *K*_a_ value for the binding to CβG20 was a little larger than that to CβG19 (Table 1). The *K*_a_ value for the binding to LβG121 was significantly smaller than those to the series of CβGs (Table 1).

The signs of both Δ*H* and Δ*S* were negative for all the binding of Chy400_4166 to the cyclic and linear β‐1,2‐glucans (Table 1). Because the observed negative Δ*H* was found to be favorable and the observed negative Δ*S* was unfavorable for binding, the binding of Chy400_4166 to the cyclic and linear β‐1,2‐glucans was driven by the favorable negative Δ*H* (Table 1). The favorable negative Δ*H* may be contributed by hydrogen bonding and CH‐π stacking between Chy400_4166 and each of the cyclic and linear β‐1,2‐glucans revealed from the X‐ray crystallographic structures of the complexes composed of Chy400_4166 and each of the β‐1,2‐glucans as described below. The magnitude of the favorable negative Δ*H* was similar among all the binding of Chy400_4166 to the cyclic and linear β‐1,2‐glucans, although that was a little smaller for the binding to CβG20 (Table 1).

On the other hand, the release of the structured water molecules surrounding Chy400_4166 and the cyclic and linear β‐1,2‐glucans upon the complex formation of Chy400_4166 with the cyclic and linear β‐1,2‐glucans contributed to a positive change in dehydration entropy. However, we observed the unfavorable negative Δ*S* in this study (Table 1). Another factor counteracted the dehydration effect and contributed to the unfavorable negative Δ*S*. A negative conformational entropy change derived from the conformational restraint upon the complex formation of Chy400_4166 with the cyclic and linear β‐1,2‐glucans may be responsible for the unfavorable negative Δ*S*. Therefore, the conformation of Chy400_4166 and the cyclic and linear β‐1,2‐glucans may be significantly restricted upon the complex formation with the unfavorable negative Δ*S*. When the number of DP for the CβG was increased, the magnitude of the unfavorable negative Δ*S* for the binding to the CβG was increased, although the magnitude of the unfavorable negative Δ*S* for the binding to CβG20 was significantly smaller than those to other CβGs (Table 1).

### Overall structures of Chy400_4166 with β‐1,2‐glucans

We successfully obtained complex structures of Chy400_4166 with LβG121, CβGs with DP 17–20 at 1.27–1.95 Å‐resolutions (Table S1). In accordance with the domain architecture specified in LiSO‐BP, Chy400_4166 is composed of two domains separated by a hinge region: domain I (66–178 aa and 336–396 aa), domain II (181–333 aa and 400–486 aa), and a hinge region (179–180 aa, 334–335 aa, and 397–399 aa) (Fig. 3A). These complexes exhibit nearly identical overall structures (root mean square deviation, RMSD < 0.2 Å). However, notable deviations are observed when compared to a predicted ligand‐free structure (RMSD ≈ 1.1 Å). This difference is attributed to the domain‐closure motion induced by substrate binding. As aforementioned in the thermodynamic analyses section, the unfavorable negative Δ*S* observed by the ITC (Table 1) may be derived from the conformational restraint upon the complex formation. The structural difference may result in the observed negative Δ*S*. When domains I of the ligand‐free structure is superimposed onto the complex with CβG17, rotation of domain II is observed visually (Fig. 3B). Analysis using DynDom [34] showed that the rotation angle was 8.5°, which is smaller than that of LiSO‐BP (24.5°). Structural comparison reveals that LiSO‐BP adopts a more closed conformation than Chy400_4166 upon substrate binding, whereas both domains in their respective ligand‐free structures maintain comparably open conformations (Fig. 3C).

**Fig. 3:**
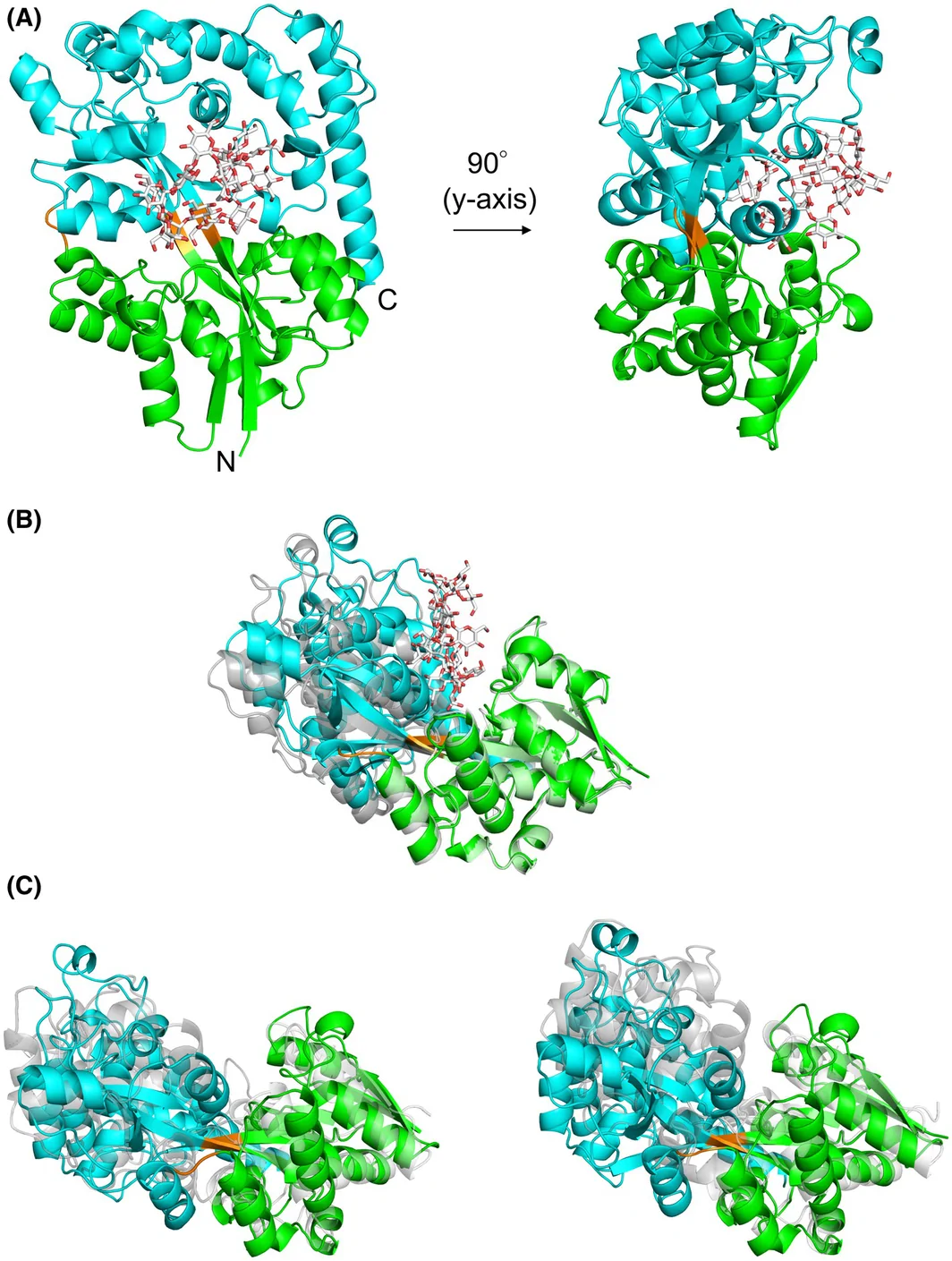
Overall structures of Chy400_4166. Domain I, domain II, and hinge region of Chy400_4166 complexed with CβG17 are shown as green, cyan, and orange cartoons, respectively. (A) Overall structure of the complex with CβG17. (B) Superimposition of the complex (Chy400_4166‐CβG17) and the predicted ligand‐free Chy400_4166. Domains I, II, and hinge regions are shown as white, gray, and orange cartoons, respectively. Both proteins are superimposed based on domain I. (C) Comparison of the overall structures of Chy400_4166 and LiSO‐BP; (left) ligand‐free structures and (right) complex structures. Domains I, II, and the hinge regions of the LiSO‐BP structures are shown as white, gray, and orange cartoons, respectively. PDB IDs of the ligand‐free structure and the complex with Sop_5_ are 5YSB and 5YSF, respectively. Color usage for displaying the predicted ligand‐free Chy400_4166 is the same as the Chy400_4166‐CβG17 complex. PyMOL was used for structural representation.

### Binding modes of β‐1,2‐glucans

Electron densities of LβGs were clearly observed as a semicircular shape in the binding pocket although the electron density of the reducing end glucose unit is somewhat weaker (Fig. 4A). The observed glucose units in the LβG complex are labeled units A–N, beginning with the unit at the nonreducing end. In the complexes of CβG18 and CβG20, electron densities of units A–J and units A–K were observed, respectively, both of which closely resemble the binding mode observed in the LβG complex (Fig. 4A). Around the W292 residue, electron densities appear to fit with the glucose units in these three complexes. However, a glucose unit is fitted only in the LβG complex because the orientations of glucose units could not be unambiguously determined in the other two complexes (Fig. 4B). In the complexes of CβG17 and CβG19, the electron density was sufficiently clear to allow for modeling the entire CβG molecules, in contrast to the other CβG complexes (Fig. 4A).

**Fig. 4:**
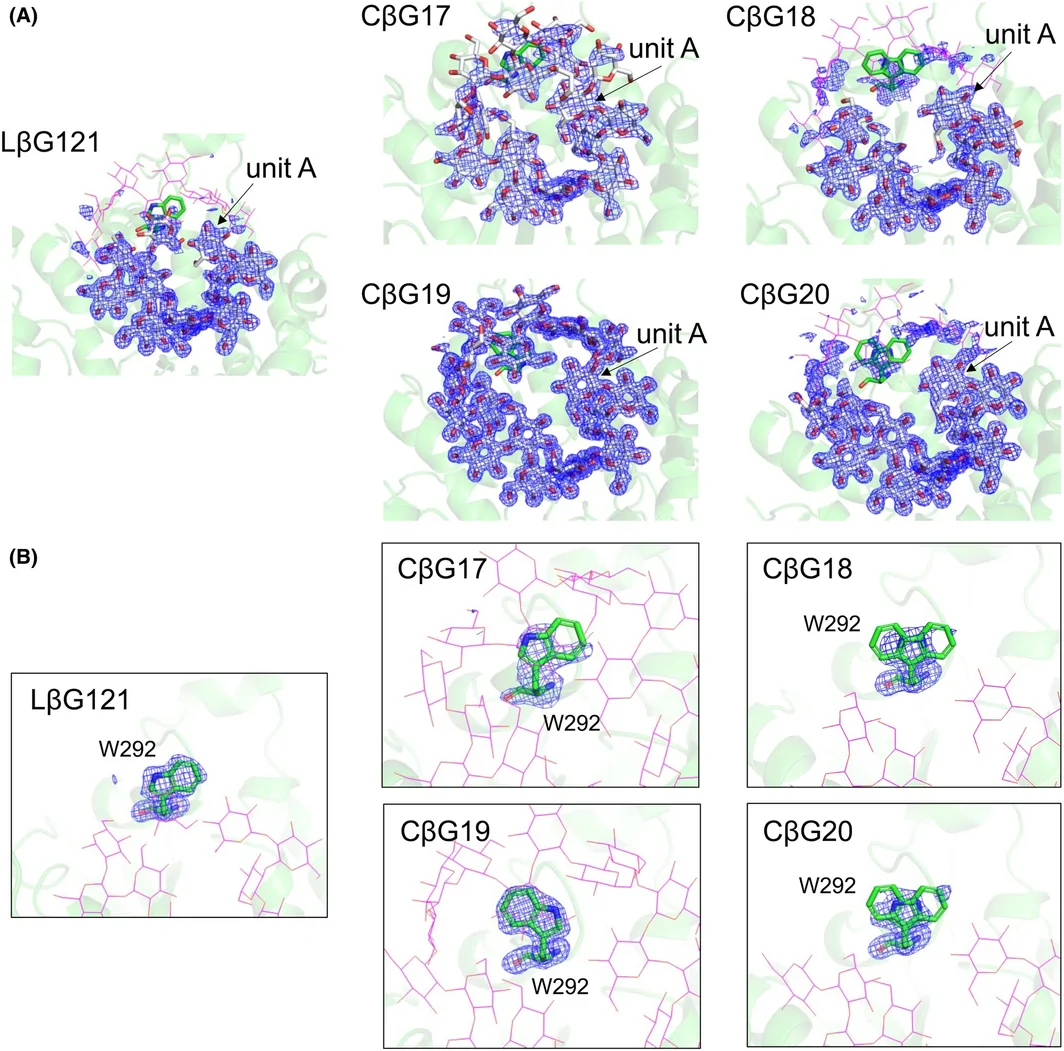
Electron densities of the substrates and W292 in the complexes of Chy400_4166. The *F*_o_ − *F*_c_ omit maps for LβG121, CβG17, CβG18, CβG19, and CβG20 (A) and W292 (B) are shown at the 2.5σ contour level and represented with blue meshes. The main chains are shown semitransparently as green cartoons. (A) Substrates are shown as white sticks. In the complexes with LβG121, CβG18, and CβG20, superimposed CβG17 molecules are shown as magenta lines. (B) The W292 residue in each complex is indicated by a green stick. The substrates are indicated by the magenta lines. PyMOL was used for structural representation.

The five complex structures are superimposed to understand the structure–function relationships. When an observed glucose unit at the nonreducing end in the complex with LβG is designated as unit A, units B–I exhibit high structural overlap across all of the complexes (Fig. S2). The glucose units at units A and J in the complex of CβG17 deviate slightly from the counterparts in the other complexes (Fig. S2). These observations suggest that units A–J constitute the fundamental moiety required for binding of β‐1,2‐glucans. This region is recognized by approximately 15 residues.

One noticeable feature is that unit G is firmly recognized by W74, W308, D336, and K419 (Figs 5A,B, 6, 7A). W74 and D336 are completely conserved among the selected Chy400_4166 homologs (Figs S3, S4). In several homologs, W308 is substituted with tyrosine residues. Nevertheless, their functions are considered to be conserved, as W308 forms a CH‐π interaction through its aromatic ring, which is also found in tyrosine residues. In addition, K419 is conserved among close Chy400_4166 homologs in Groups 1 and 2. Taken together, these observations suggest that unit G and the four residues are essential for substrate recognition.

**Fig. 5:**
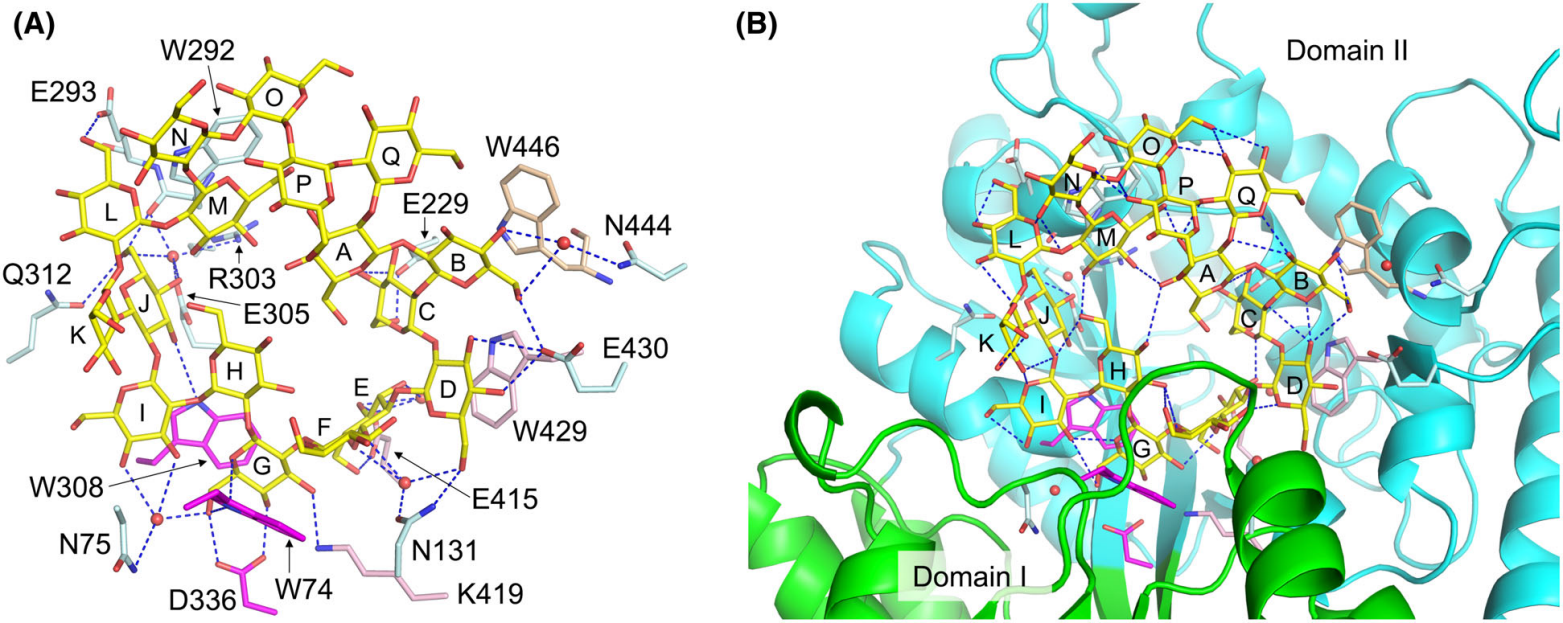
Recognition of CβG17. (A) Residues recognizing CβG17. Hydrogen bonds, including those mediated by one water molecule, are shown as blue dashed lines. Each glucose unit is labeled with the defined unit name. The residues are colored based on chemical conservation level: magenta (conserved within Groups 1–3), light pink (conserved within Groups 1 and 2), light blue (not conserved). (B) Intramolecular interaction of CβG17. The main chain is added as a cartoon to (A). Domains I and II are shown in green and cyan, respectively. Intramolecular hydrogen bonds are shown as blue dashed lines instead of intermolecular ones in (A). PyMOL was used for structural representation.

**Fig. 6:**
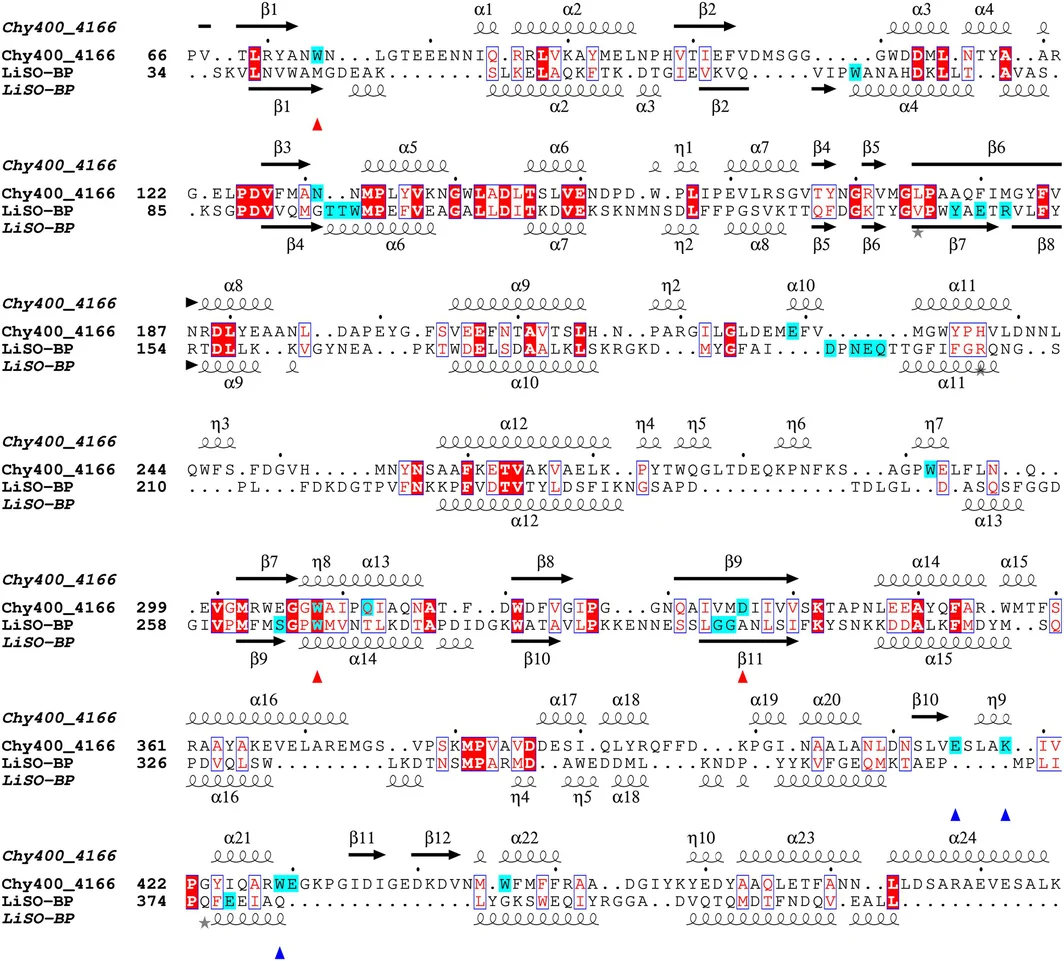
Structure‐based amino acid sequence alignment of Chy400_4166 and LiSO‐BP. Structure‐based alignment was performed using ligand‐free structures of Chy400_4166 and LiSO‐BP (PDB IDs: 22HH and 5YSF, respectively) on the SALIGN server (https://modbase.compbio.ucsf.edu/salign/) [35]. Substrate recognition residues are highlighted in cyan. Red and blue triangles indicate highly conserved residues in Groups 1–3 and 1–2, respectively. The figure was prepared using ESPript3.2 (https://espript.ibcp.fr/ESPript/ESPript/index.php) [36].

**Fig. 7:**
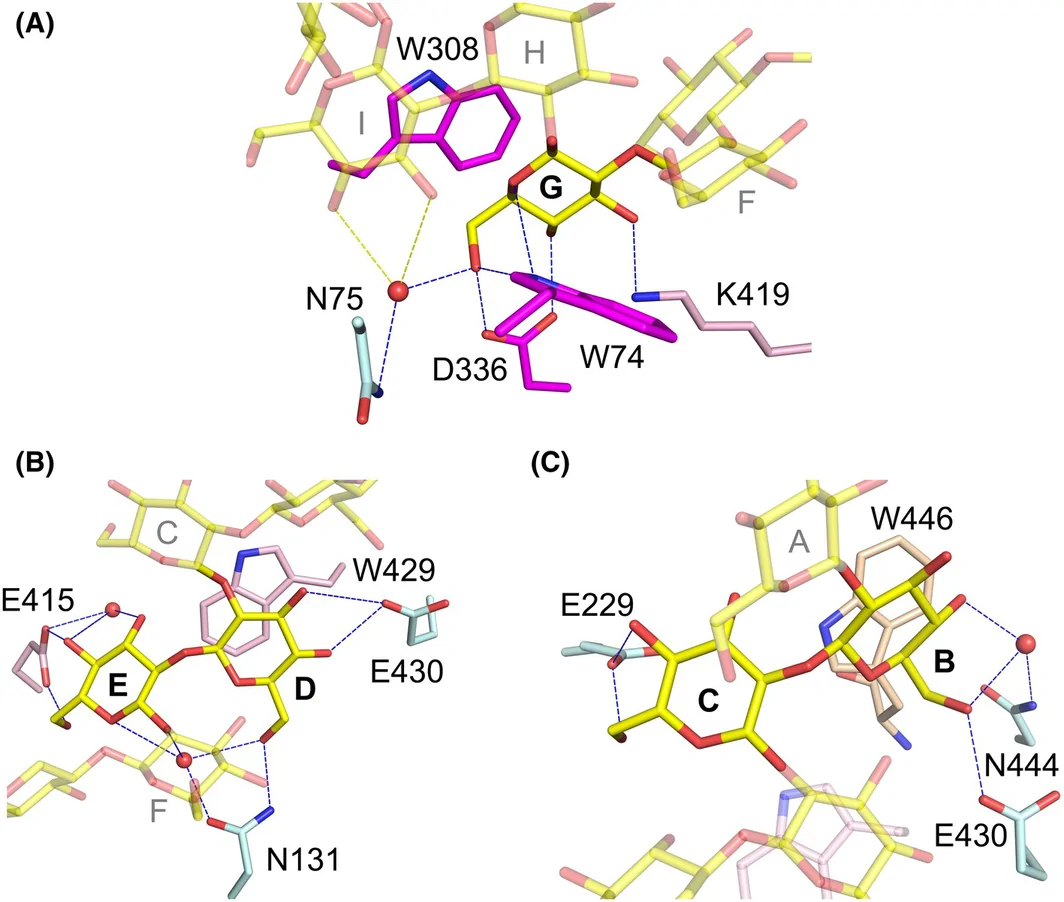
Close‐up view of recognition of glucose units in Chy400_4166‐CβG17 complex. (A–C) Recognition of unit G (A), units D and E (B), and units B and C (C). The residues are colored based on chemical conservation level: magenta (conserved within Groups 1–3), light pink (conserved within Groups 1 and 2), light blue (not conserved). Unfocused glucose units and a residue are displayed semitransparently. PyMOL was used for structural representation.

E415 and W429 are conserved residues with chemically similar residues in Groups 1 and 2 (Figs S3, S4). E415 forms bidentate hydrogen bonds with unit E and W429 forms CH‐π stacking interaction with unit D (Fig. 7B). Therefore, units D and E are important for substrate recognition.

Q229, E430, and W446 participate in the recognition of units B–D (Fig. 7C). This appears to be firm recognition. W446 is a highly conserved residue (Figs S3, S4). On the opposite side of the units in the substrate pocket, unit I is recognized by N75 (water‐mediated) and W308 (Fig. 5A,B). Unit J forms hydrogen bonds with W308, Q312, R303, and E305 through a water molecule. The substitution of W308 with a tyrosine residue found in Group 1 likely disrupts the hydrogen bond at this position. Moreover, the recognition residues of units B–D, I, and J are conserved within a limited range in Group 1. Such conservation patterns may be involved in their preference for chain length and substrate form (cyclic or linear). In addition, several units, such as A and H, are not recognized by any residue, which may be compensated for by intramolecular interactions (Fig. 5B).

Units K and beyond are observed only in the complexes with CβG17 and CβG19 (Fig. 4). This region is less recognized than units A–J. Remarkable features of the binding modes of CβGs are observed around W292. W292 forms a CH‐π stacking interaction with unit M in CβG17 (Fig. S5). The same residue also forms the same type of interaction with unit O in CβG19. However, the indole ring of W292 is flipped between these states, leading to a significant deviation in the positions of the corresponding glucose units between the two complexes. Unit K in CβG17 potentially collides with the W292 side chain in the complex with CβG19. Whereas, unit Q in CβG19 forms hydrogen bonds with a nitrogen atom in the indole ring of W292 and potentially collides with the W292 side chain in the complex with CβG17. This observation suggests that the flipping of the side chain in W292 allows the accommodation of CβGs with different DPs with stronger affinity. In the complexes with CβG18 and CβG20, the electron densities of the six‐membered ring moieties in the side chains of W292 are disordered (Fig. 4B), thus suggesting conformational flexibility at this position. The fact that the glucose units other than units A–J are almost disordered suggests that there are multiple binding modes for CβG18 and CβG20. Given that the electron densities facing W292 are unmodeled but could be fitted with glucose units (Fig. 4A), it is suggested that W292 participates in the binding of CβG18 and CβG20.

Here, we correlate the structural findings with the thermodynamic results presented in the thermodynamic analyses section in order to better understand the binding mechanism. The unfavorable negative Δ*S* observed by the ITC (Table 1) may be derived from the conformational restraint upon the complex formation of Chy400_4166 with β‐1,2‐glucans. Judging from the smaller magnitude of the negative Δ*S* for CβG20 in comparison with that for CβG18, CβG20 may bind to Chy400_4166 with a more flexible conformation around W292 than CβG18. In the case of LβGs, a glucose unit is fitted in the electron density at the vicinity of W292. As aforementioned in the thermodynamic analyses section, the favorable negative Δ*H* observed by the ITC (Table 1) may be derived from hydrogen bonding and CH‐π stacking between Chy400_4166 and β‐1,2‐glucan. Together with the result that the magnitudes of the negative Δ*H* for the binding of CβG17–19 and LβG121 were similar, it is considered that W292 contributes to the binding affinity for LβGs as well. The magnitude of the unfavorable negative Δ*S* for the binding of LβG121 is restored from that for CβG20 (Table 1), which is consistent with the speculation that constraint when binding at units A–J is stronger for LβG121 than CβG20.

## Discussion

### Roles of Chy400_4166

In this study, we have found that Chy400_4166 bound to β‐1,2‐glucans with both linear and cyclic forms, and complex structures with LβGs and CβGs were obtained. This is the first found β‐1,2‐glucan binding protein of type I ABC transporter. LiSO‐BP is a Sop_n_s‐binding protein but also shows weak affinity for LβGs [30]. However, this affinity is likely too weak to be physiologically relevant. The glucans bound to Chy400_4166 adopt rounded shapes, regardless of the linear and cyclic forms. In addition, W292 and W446, which are optionally positioned based on sequence analysis, are likely to orient both ends of the substrate to fit cyclic forms. Given that β‐1,2‐glucans primarily exist in cyclic forms in nature, it is suggested that the protein is fundamentally specialized for incorporation of CβGs but simply lacks a mechanism to exclude LβGs as substrates.

In the Chy400_4166 (Chy400_4165–4178) gene cluster, there was a series of putative β‐1,2‐glucan‐associated proteins (Fig. 8). Chy400_4174 and 4176 are SGLs ([15] and data not shown, respectively) with and without an *N*‐terminal signal peptide, respectively. Chy400_4165 is a putative SOGP because it is distantly related to characterized GH94 SOGP from *L. innocua* and *L. phytofermentans* [14, 25]. Chy400_4178 is considered to be a β‐glucosidase preferring Sop_n_s because substrate recognition residues important for substrate preference in β‐1,2‐glucan‐associated β‐glucosidases from *L. innocua* and *B. thetaiotaomicron* are conserved in Chy400_4178 [29, 37]. Chy400_4177 is a homolog of a GH35 transglycosylase (IALB_1185 from *Ignavibacterium album*) that disproportionates Sop_n_s by transferring a glucose unit [26]. IALB_1185 acts on various glucosides as acceptors, rather than Sop_n_s. Together with the fact that a gene cluster of IALB_1185 does not contain a β‐glucosidase, IALB_1185 may play a role in producing sophoroside in the gene cluster [26]. However, given that the gene cluster of Chy400_4165–4178 contains GH3 β‐glucosidase, disproportionation is considered to be an actual role in metabolism of β‐1,2‐glucans. Chy400_4165, 4177, and 4178 have no *N*‐terminal signal peptides, suggesting that they are cytosolic enzymes as well as Chy400_4176. Overall, if β‐1,2‐glucans are incorporated into the cytosol, these compounds can be degraded to glucose for biochemical functions. Considering that ABC transporters are usually located at inner membrane in Gram‐negative bacteria, *C. aurantiacus* may have another system transporting β‐1,2‐glucans into periplasmic space through outer membrane.

**Fig. 8:**
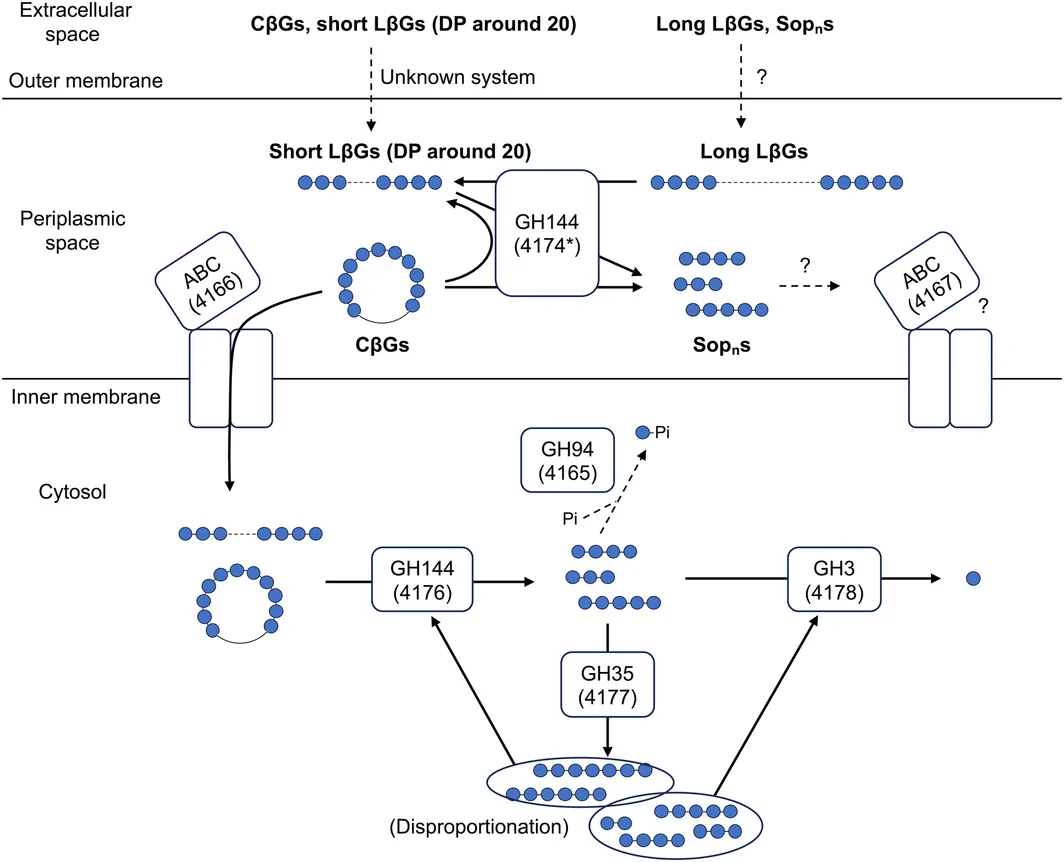
Speculated metabolic pathway of linear and cyclic β‐1,2‐glucans. glucose units are represented by blue circles. Solid arrows indicate that the reactions are presumed to be based on biochemically characterized homologs. Chy400_4165 are presumed as a glycoside phosphorylase producing α‐glucose 1‐phosphate. However, substrates of Chy400_4165 have not been sufficiently presumed based on previous studies. The presumed reaction of Chy400_4165 is indicated by a dashed arrow. The substrate‐binding subunits of ABC transporters and the proteins annotated as GH enzymes are boxed with rounded rectangles. Parentheses indicate the numbers in locus tags. An asterisk attached to 4174 (Chy400_4174) indicates its potential to function in the extracellular space. The compartments of a cell of *C. aurantiacus* Y‐400‐fl are labeled on the left. Question marks indicate that the presence of substrate uptake and binding systems is currently unknown.

### Structural comparison with LiSO‐BP

LiSO‐BP is a binding protein of Sop_n_s with DP3 or higher but shows weak affinity to LβGs unlike Chy400_4166 [30]. Therefore, we performed a structural comparison of the two proteins. LiSO‐BP binds to the nonreducing end of a Sop_n_ molecule, which is oriented perpendicular to the arrangement of the three hinge regions between the domains (shown in orange, Fig. 9A, left). This orientation of the substrate results in exo‐type binding of LiSO‐BP. In contrast, in Chy400_4166, the substrate is bound in an orientation parallel to the corresponding arrangement of the hinge regions (shown in orange, Fig. 9A, right). Chy400_4166 binds to a middle region of a β‐1,2‐glucan molecule, which is consistent with the stoichiometry (*N* > 2) determined by ITC in the presence of LβG121 (Table 1). If Chy400_4166 can bind to a LβG chain in an exo‐manner, the stoichiometry should be *N* = 1, regardless of DP of the chain. These observations have indicated that the binding mode of Chy400_4166 is endo‐type.

**Fig. 9:**
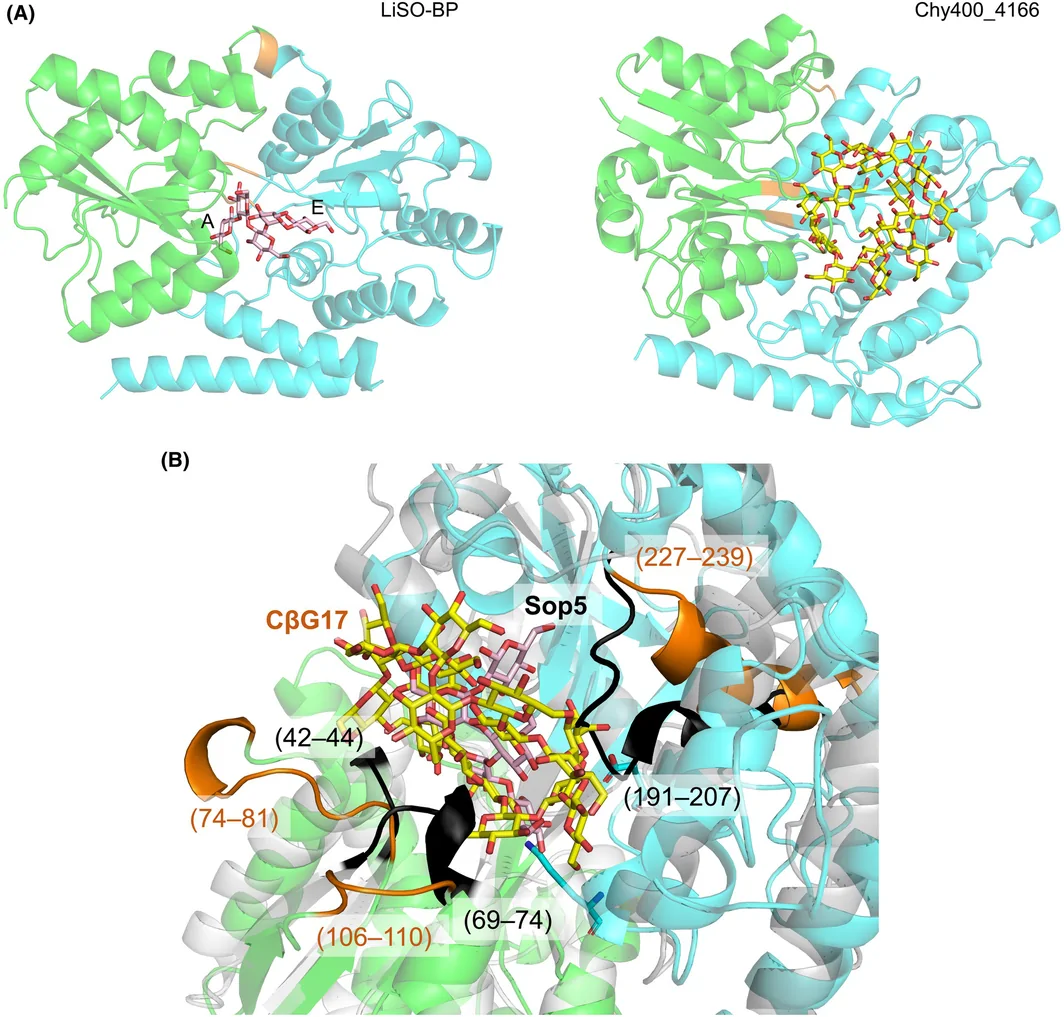
Comparison of complexes of Chy400_4166 with CβG17 and LiSO‐BP with Sop_5_. (A) Domains I, II, and the hinge regions in both proteins are shown as green, cyan, and orange cartoons, respectively. CβG17 and Sop_5_ are shown as yellow and light pink sticks, respectively. (B) Superimposition of Chy400_4166‐CβG17 and LiSO‐BP‐Sop_5_. The colors of domains I, II, and the hinge regions of LiSO‐BP are changed to white, white, and gray, respectively. Cartoons are displayed semi‐transparently. Residues 74–81, 106–110, and 227–239 in Chy400_4166 and residues 42–44, 69–74, and 191–207 are colored in orange and black, respectively. PyMOL was used for structural representation.

The complex structures of LiSO‐BP with Sop_n_s adopt more closed conformations than those of Chy400_4166 (Fig. 3C, right). Furthermore, regions around 45 aa, 70 aa, and 195 aa protrude into the pocket of LiSO‐BP, narrowing the pocket (Fig. 9B). In Chy400_4166, these regions are located away from the pocket. These structural differences allow Chy400_4166 to accommodate larger substrates such as CβGs.

In LiSO‐BP, chain‐length specificity is clearly revealed. LiSO‐BP, as well as Chy400_4166, is composed of two domains (domains I and II) (Fig. 10A). The two domains close when capturing a substrate (Sop_n_ with a DP of 3 or higher). This capturing system is known as the Venus‐fly trap mechanism [38]. In the LiSO‐BP‐Sop_5_ complex, units A and B are recognized mainly by residues in domain I, whereas the glucose moieties at units C and beyond are recognized by residues in domain II [30]. The glucose moieties in units A–C are common binding sites between the complexes with Sop_3_, Sop_4_, and Sop_5_ (Fig. 10B). Thus, unit C is key to chain‐length specificity. A comparison between the ligand‐free structure and the complex with Sop_5_ suggests that D193 and Q197 in domain II approach unit C in Sop_5_ for interaction (Fig. 10B). The closure motion is important for chain‐length specificity. In addition, E45 and W268 are appropriately arranged to recognize unit B by closure motion.

**Fig. 10:**
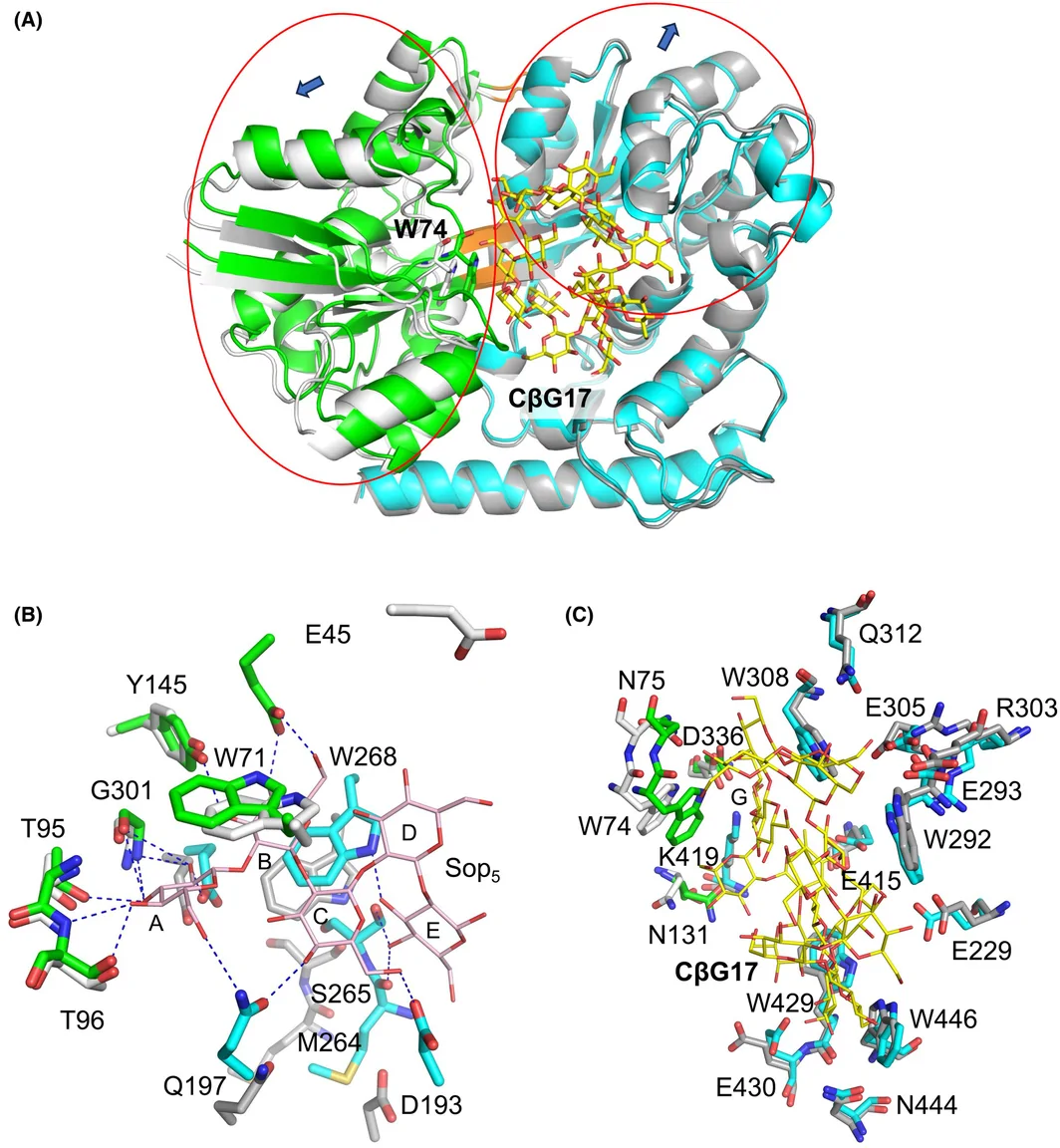
Superimposition of ligand‐free and complex structures. (A) Superimposition of the overall structures of Chy400_4166. Domains I, II, and the hinge regions in the complex structure are shown as a cartoon in green, cyan, and orange, respectively. Domain I and hinges, and domain II in the ligand‐free structure are shown as a cartoon in white and gray, respectively. Superimposition is performed using domain II. Red dashed circles highlight clusters moving in the same directions. The directions are indicated with light blue arrows. CβG17 is shown as a yellow stick. W74 residues in the complex and the ligand‐free structures are shown as sticks. (B, C) Superimposition of the substrate recognition residues in LiSO‐BP (B) and Chy400_4166 (C). Sop_5_ (B) and CβG17 (C) are shown as thin yellow and light pink sticks, respectively. The defined glucose unit names are labeled [partially in (C)]. (B) Residues of domains I and II in the complex structure are shown as green and cyan sticks, respectively. In the case of the ligand‐free structure, domains I and II are shown as white and gray sticks, respectively. (C) Color usage of residues is based on (A). PyMOL was used for structural representation.

Unlike LiSO‐BP, Chy400_4166 possesses many substrate recognition residues in domain II. Furthermore, the apparent motions of the substrate recognition residues are overall smaller than those in LiSO‐BP (Fig. 10B,C). Among these residues, W74 and N75 shift in the direction in which the substrate pocket expands in the predicted ligand‐free structure. W74 is a highly conserved residue among Chy400_4166 homologs (Fig. S4). This shift can cause W74 to lose its hydrogen bond interactions with the substrate. Hydrophobic interactions with W74 are potentially reduced in the open conformation. Thus, W74 is considered a key residue for the induced fit.

### Concluding remarks

The structural and functional analyses of Chy400_4166 revealed that, despite both Chy400_4166 and LiSO‐BP targeting glucose polymers with identical linkages, their binding modes differ dramatically based on the chain lengths of their substrates. It is intriguing in that this finding implies the remarkable diversity among β‐1,2‐glucan‐associated binding proteins. Furthermore, the discovery of this novel β‐1,2‐glucan transport system provides a crucial stepping stone toward understanding the relevance of β‐1,2‐glucan, which is rare yet widespread in nature.

## Methods

### Materials

LβG121 was prepared based on several references [15, 16, 39]. The number represents an average DP calculated based on number‐averaged molecular weight. CβGs, prepared according to the method of Hisamatsu *et al*. [40], were kindly provided by Prof. Okada. The other polysaccharides used for Native‐PAGE were laminarin (Merck, NJ, USA), lichenan (Neogen, MI, USA), barley β‐glucan (Neogen), tamarind xyloglucan (Neogen), beechwood xylan, carboxymethyl (CM) curdlan (Neogen), CM cellulose (Merck), arabinan (Neogen), arabinogalactan from Larch Wood (Tokyo Chemical Industry, Tokyo, Japan), guar gum (FUJIFILM Wako Pure Chemical Corporation, Osaka, Japan). Sop_n_s were prepared based on Nakajima *et al*. [14].

### Cloning, expression, and purification of Chy400_4166

The genomic DNA of *C. aurantiacus* DSM637 was purchased from the Leibniz Institute DSMZ (Leibniz, Germany). Primers used for subcloning the gene *chy400_4166* were 5′–GTTGAGTGGACatatgGGCTGTACATCAGC–3′ (forward primer) and 5′–CCTTACGTGATCCctcgagCTTCAATGCCGATTC–3′ (reverse primer).

The primer pair for subcloning of the gene *chy400_4166* was designed to exclude an *N*‐terminal signal region. The signal region was predicted using SignalP4.0 [41]. Restriction sites of NdeI and XhoI (underlined) were introduced in the forward and reverse primers, respectively, to fuse *C*‐terminal His_6_‐tag derived from a vector. The gene was amplified by PCR reaction using KOD plus (Toyobo, Japan). After the amplified product and pET30a were digested with NdeI and XhoI (Takara Bio, Japan), the amplified product was inserted into the digested vector using DNA ligase (Ligation high ver.2, Toyobo). After the sequence of the inserted region was checked by outsourcing (Hokkaido System Science, Co., Ltd., Japan), the plasmid was transformed into *E. coli* strain Rosetta2(DE3). The transformant was incubated in LB medium including kanamycin (30 μg·mL^−1^) until OD_600_ reached around 0.8. Isopropyl 1‐thio‐β‐d‐galactopyranoside (0.1 mm as a final concentration) was added, and then, induction was performed at 30 °C for 21 h with sufficient agitation. The transformant cells were collected by centrifugation at 8800 × ***g*** for 10 min and then suspended in 50 mm Tris/HCl buffer (pH 7.5). The suspended cells were disrupted by sonication, and the cell debris was removed by centrifugation at 27,700 × ***g*** for 15 min to obtain cell extracts. The cell extract was applied to a HisTrap™ FF crude 5 mL column (Cytiva, MA, USA) pre‐equilibrated with equilibration buffer (50 mm Tris/HCl, 500 mm NaCl, pH 7.5). Unbound proteins were removed by washing with the equilibration buffer containing 10 mm imidazole, and then, the adsorbed proteins were eluted with the equilibration buffer containing 400 mm imidazole. The eluate was concentrated and buffer‐exchanged to 20 mm sodium phosphate buffer (pH 6.99) with Vivaspin Turbo 15 10000 MWCO (Sartorius, Goettingen, Germany). The purity was estimated by SDS/PAGE. For crystallization, the buffer was exchanged to 5 mm MOPS (pH 7.0). The purified protein migrated as a single band with 50 kDa on an SDS/PAGE gel, which is consistent with the theoretical molecular mass of the recombinant Chy400_4166 (52934.92). Protein concentration was determined by measurement of the absorbance at 280 nm and calculation from the theoretical extinction coefficients of Chy400_4166 (molar extinction coefficient is 103,945 A_280_·mol^−1^·cm^−1^).

### Native‐PAGE

Native‐PAGE was performed using 8% acrylamide gel without SDS in the whole system of SDS/PAGE. Each polysaccharide (0.1% as a final concentration) was included in a separation gel. Electrophoresis was performed at a constant current of 30 mA, with the voltage limited to 70 V. The voltage was set to a lower value as needed. Gels were cooled with ice during electrophoresis. DynaMarker Protein MultiColorIII (BioDynamics Laboratory Inc., Tokyo, Japan) was used as a protein marker. Bovine serum albumin (3 μg, FUJIFILM Wako Pure Chemical Corporation) was used as a negative control. Purified Chy400_4166 (5 μg) was applied to each well.

### Crystallography

Initial screening of crystallization conditions was performed using PACT premire™HT‐96 and JCSG‐plus™HT‐96 (Molecular Dimensions, UK) and 8.7 mg·mL^−1^ of Chy400_4166 containing 1% LβG121. Reservoir solution (1 μL) and protein solution with the polymer (1 μL) were mixed, and then, the mixture was incubated at 20 °C in a plate for sitting‐drop vapor diffusion method (96‐wells CrystalQuick plates, Greiner Bio‐One, Germany). The final solutions used for hanging‐drop vapor diffusion method were as follows: reservoir solution, around 2.0 M ammonium sulfate and bis‐Tris (pH 5.6); the protein solution with linear or cyclic β‐1,2‐glucan (approximately 9 mg·mL^−1^ and 1%, respectively). After drops were prepared by mixing the reservoir (1 μL) and the protein solution (3 μL), the drops were incubated at 20 °C in a VDX plate (Hampton Research, CA, USA). The crystals were cryoprotected by soaking the reservoir solution supplemented with 30% glycerol and 1% linear or cyclic β‐1,2‐glucan before data collection. Data collection was performed at 100 K in a nitrogen‐gas stream. The initial phase information was obtained by molecular replacement using a structure predicted by AlphaFold2 [42, 43] as a model. ‘The model is available in ModelArchive (https://modelarchive.org/) at https://www.modelarchive.org/doi/10.5452/ma‐72ttk’. A model building was performed using ArpWarp and Buccaner, and then, automated and manual refinement was performed using refmac5 and coot, respectively [44, 45, 46, 47]. The final structures were validated when deposited to wwPDB One Dep System (https://deposit‐1.wwpdb.org/). The obtained structures were visualized by PyMOL (https://pymol.org/).

### Isothermal titration calorimetry (ITC)

We conducted ITC analyses by the Microcal ITC200 system (Malvern Inc., UK). We prepared Chy400_4166 solution through extensive dialysis in 20 mM sodium phosphate buffer (pH 7.0) and dissolved a substrate in the dialysis buffer. We injected Chy400_4166 solution 20 times in 2 μL increments at 3 min intervals and 25 °C from the ITC injection syringe rotating at 1000 rpm into the ITC sample cell containing the substrate solution (effective volume = 200 μL) without changing the experimental conditions. We thoroughly degassed all the sample solutions before loading them into the ITC sample cell to avoid the possible bubble formation during titration. In the control experiments, we titrated Chy400_4166 solution into the dialysis buffer under the same experimental conditions to determine the heat of dilution. We subtracted the heat of dilution of the injectant from that of each injection. We analyzed the corrected heat by the Microcal Origin software, according to the manufacturer's instructions. Each corrected heat was divided by the number of Chy400_4166 moles injected and plotted as a function of the [Chy400_4166]/[substrate] molar ratio. We analyzed the plot according to a model for one set of binding sites using the above‐described software. The reported thermodynamic parameters are an average of three individual measurements.

### Sequence analysis

Sequences were extracted by a BLAST search against the KEGG database (https://www.kegg.jp/kegg/kegg2.html) [48] using Chy400_4166 as a query. The top‐ranked homologous sequences were used for multiple sequence alignment and preparation of the phylogenetic tree by MUSCLE (https://www.ebi.ac.uk/jdispatcher/msa/muscle?stype=protein) [49].

## Author contributions

MN and HN conceived the study; MN and HT planned experiments; KK, TK, RH, NT, and HT performed experiments; KK, TK, RH, and HN prepared the purified proteins and the materials for biochemical experiments; TK and RH performed protein crystallography; MN performed the phylogenetic analyses; MN and HN supervised the study, and MN and HT drafted the manuscript. All authors reviewed the final version of the manuscript.

## Conflict of interest

The authors declare no conflict of interest.

## Supporting information


Data S1.
**Table S1.** Data collection and statistics.
**Fig. S1.** SGL gene cluster in *C. aurantiacus* Y‐400‐fl.
**Fig. S2.** Superimposition of substrates in the Chy400_4166 complexes.
**Fig. S3.** Phylogenetic tree of Chy400_4166 and its homologs.
**Fig. S4.** Conservation of substrate recognition residues.
**Fig. S5.** Comparison of recognition of CβG17 and CβG19 by W292.

## Data Availability

Atomic coordinates and structure factors of the crystal structures have been deposited in the Protein Data Bank under accession numbers 22HH, 22HI, 22HJ, 22HK, and 22HL.
