# Supplementary material for: Structural and thermodynamic analyses of a novel β‐1,2‐glucan binding mode in the ABC transporter solute‐binding protein Chy400_4166 from Chloroflexus aurantiacus
Source: FEBS J. 2026 May 10;293(21):6395–412. doi: 10.1111/febs.70576 (PMC13731917; doi:10.1111/febs.70576)
Supplement: Supplementary file 1 [file FEBS-293-6395-s001.pdf]

Supplementary information

Title

Structural and thermodynamic analyses of a novel β-1,2-glucan binding mode of ABC transporter solute-binding protein Chy400_4166 from *Chloroflexus aurantiacus*

Authors

Kazuya Kato[1], Tatsuya Kaneko[1], Rintaro Hirayama[1], Nobukiyo Tanaka[1], Hiroyuki Nakai[3], Hidetaka Torigoe[2*], Masahiro Nakajima[1*]

Affiliations

[1] Department of Applied Biological Science, Faculty of Science and Technology, Tokyo University of Science, 2641 Yamazaki, Noda Chiba 278-8510, Japan.

[2] Department of Applied Chemistry, Faculty of Science, Tokyo University of Science, 1-3 Kagurazaka, Shinjuku-ku, Tokyo 162-8601, Japan

[3] Faculty of Agriculture, Niigata University, 8050 Ikarashi 2-no-cho, Nishi-ku, Niigata 950-2181, Japan

* To whom correspondence should be addressed: Department of Applied Biological Science, Faculty of Science and Technology, Tokyo University of Science, 2641 Yamazaki, Noda, Chiba 278-8510, Japan; Email: m-nakajima@rs.tus.ac.jp; Tel.: +81-471-24-1501; Fax: +81-471-23-9767; Department of Applied Chemistry, Faculty of Science, Tokyo University of Science, 1-3 Kagurazaka, Shinjuku-ku, Tokyo 162-8601, Japan; E-mail: htorigoe@rs.tus.ac.jp; Tel: +81-3-5228-8259; Fax: +81-3-5261-4631

Table S1. Data collection and statistics.

Data set	Chy400_4166-LβG	Chy400_4166-CβG17	Chy400_4166-CβG18	Chy400_4166-CβG19	Chy400_4166-CβG20
**Data collection**					
Beamline	KEK BL-5A	KEK BL-5A	KEK BL-5A	KEK BL-5A	KEK BL-5A
Space group	$P2_12_12_1$	$P2_12_12_1$	$P2_12_12_1$	$P2_12_12_1$	$P2_12_12_1$
Unit cell parameters (Å)	a = 73.71<br>b = 83.26<br>c = 94.27	a = 72.39<br>b = 82.91<br>c = 94.07	a = 72.70<br>b = 82.85<br>c = 94.10	a = 72.49<br>b = 83.20<br>c = 94.24	a = 72.65<br>b = 83.21<br>c = 94.15
Resolution (Å)[a]	47.63–1.53 (1.56–1.53)	47.18–1.95 (2.00–1.95)	47.25–1.60 (1.63–1.60)	47.28–1.27 (1.29–1.27)	47.32–1.37 (1.39–1.37)
Total reflections[a]	573796 (22087)	510827 (34921)	938372 (48615)	1921722 (89926)	1540650 (76381)
Unique reflections[a]	88131 (4318)	41270 (2842)	75627 (3708)	150304 (7340)	120251 (5852)
Completeness (%)[a]	100 (100)	98.5 (97.4)	100 (99.9)	100 (100)	100 (100)
Multiplicity[a]	6.5 (5.1)	12.4 (12.3)	12.4 (13.1)	12.8 (12.3)	12.8 (13.1)
Mean $I/\sigma(I)$[a]	18.2 (2.3)	12.8 (2.4)	33.9 (4.4)	35.6 (3.5)	33.0 (3.0)
R_{merge} (%)[a]	5.3(63.7)	10.1 (94.2)	4.4 (60.8)	3.1 (78.2)	3.7 (89.5)
R_{pim} (%)[a]	3.4 (48.0)	4.3 (40.3)	1.9 (25.2)	1.3 (33.7)	1.5 (37.5)
$CC_{1/2}$[a]	(0.742)	(0.942)	(0.946)	(0.907)	(0.906)
**Refinement**					
Resolution (Å)	47.63–1.53	47.18–1.95	35.99–1.68	47.28–1.27	47.32–1.37
No. of reflections	83668	39116	71687	142677	114194
No. of atoms	3976	3655	3906	4104	3983
No. of water molecules	343	104	302	402	387
R_{work}/R_{free} (%)	15.8/17.6	24.7/28.4	16.7/18.1	16.5/18.1	16.9/18.4
No. of asymmetric units	1	1	1	1	1
RMSD from ideal values					
Bond lengths (Å)	0.0111	0.0062	0.0121	0.0132	0.0128
Bond angles (°)	1.8210	1.4773	1.9117	1.9196	1.9115
Average B-factors (Å^2)					
Protein (chain A)	19.4	32.4	21.6	18.2	19.9
Ligand					
LβG or CβG	29.2	40.9	26.5	21.9	22.5
Solvent	30.9	31.8	31.7	28.8	30.6
Ramachandran plot (%)					
Favored	96.7	96.2	96.7	96.7	96.7
Allowed	3.3	3.8	3.3	3.3	3.3
Outlier	0.0	0.0	0.0	0.0	0.0
**PDB entry**	22HH	22HI	22HJ	22HK	22HL

[a] Values in parentheses are for outer shells.

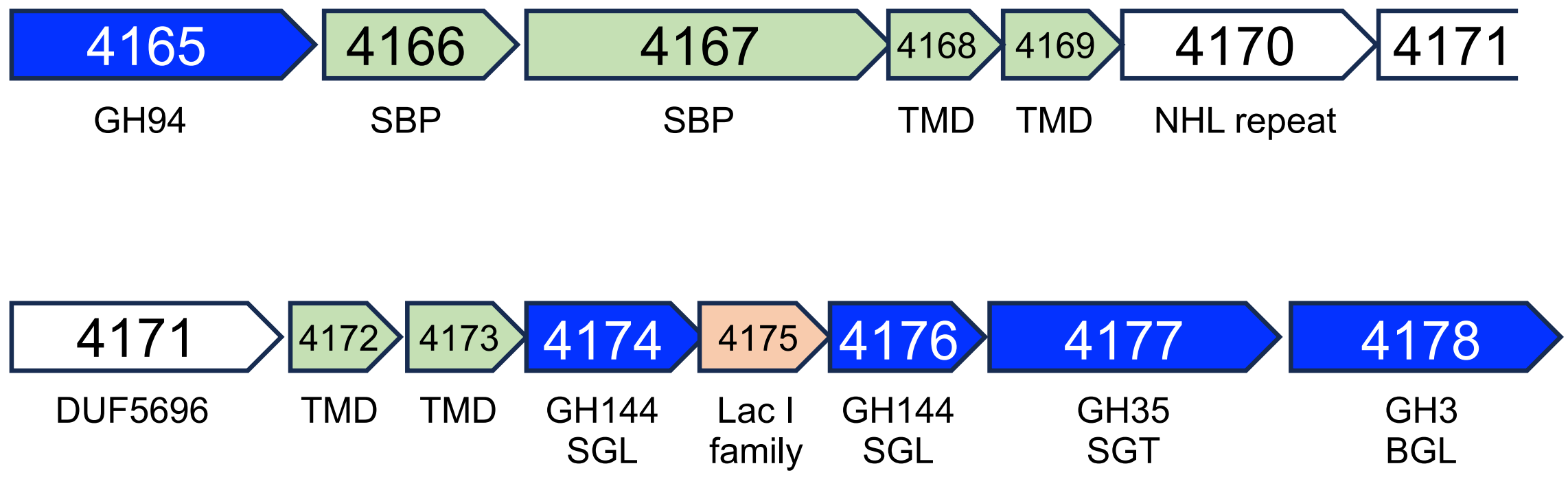

Figure S1. SGL gene cluster in *C. aurantiacus* Y-400-fl.

Arrowheads numbered with KEGG locus tags show the orientation of genes in the cluster. Annotations of GH enzymes are based on the CAZy database [20]. SGL, SGT (β-1,2-glucosyltransferase, EC2.4.1.391), and BGL are annotated according to their experimentally validated homologs. Annotations of SBPs, TMDs, Lac I family, NHL repeat, and DUF5696 are based on the KEGG (Kyoto Encyclopedia of Genes and Genomes) database (https://www.genome.jp/kegg/). Arrowheads for putative GH enzymes, ABC transporters, and Lac I family are colored in blue, light green, and salmon pink. Function unknown proteins are indicated by the white arrowheads. Arrowhead for the gene encoding Chy400_4171 is duplicated to show its relative position.

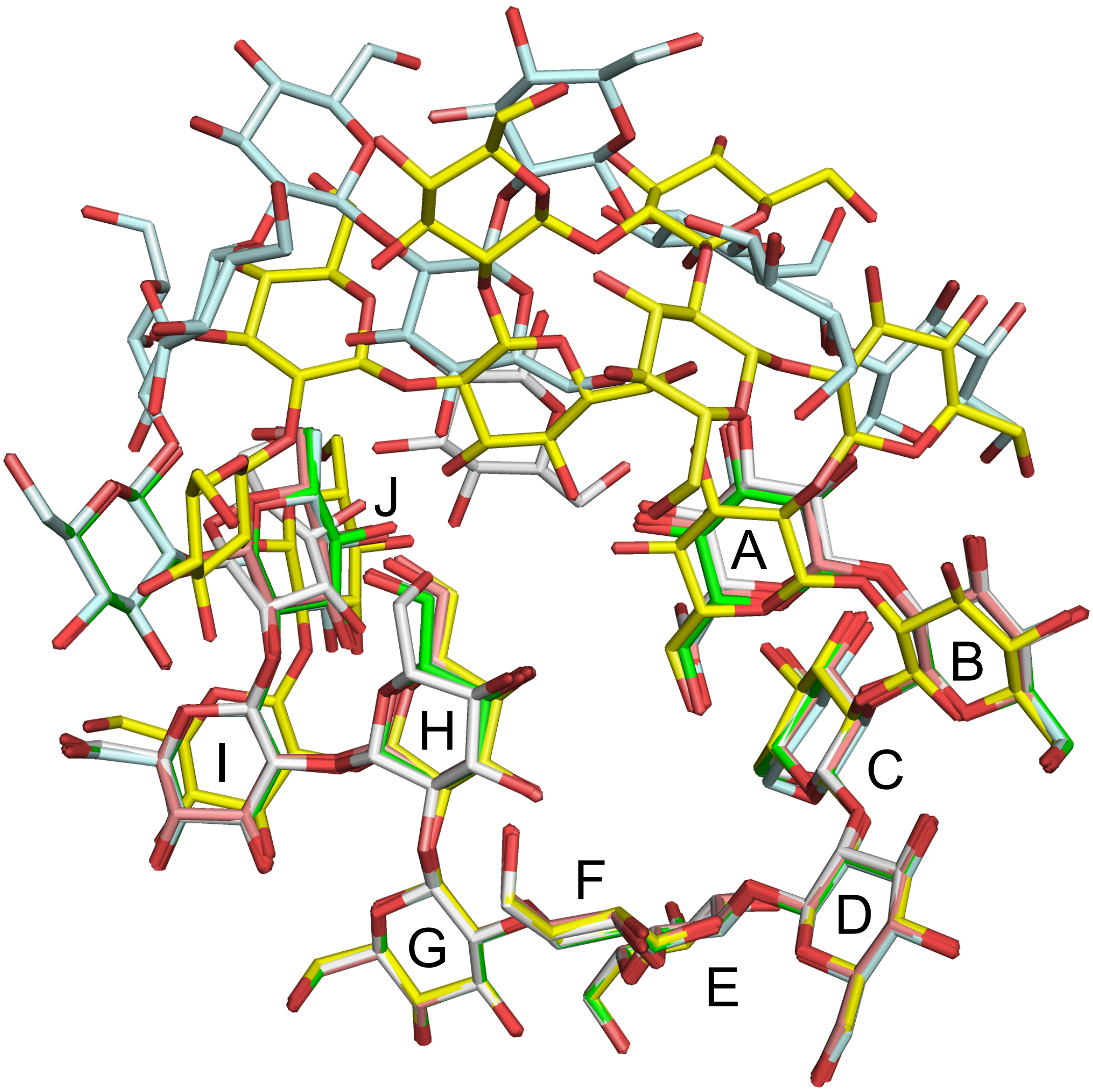

Figure S2. Superimposition of substrates in the Chy400_4166 complexes.

Substrates are indicated by sticks (white, LβG; yellow, CβG17; green, CβG18; pale cyan, CβG19; light pink, CβG20). The defined unit names are labelled at units A–J. PyMOL was used for structural representation.

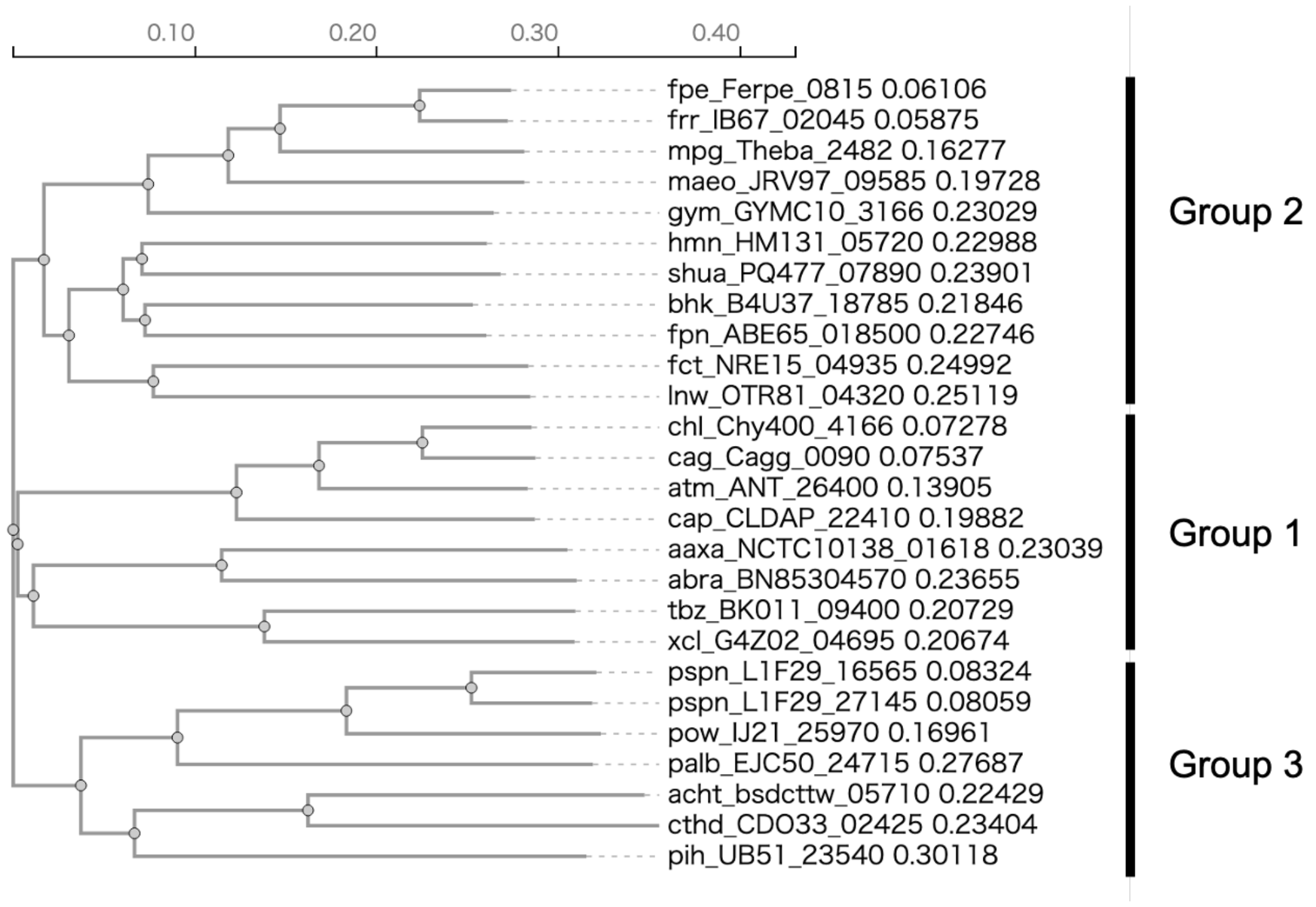

Figure S3. Phylogenetic tree of Chy400_4166 and its homologs.

Sequences were extracted by a BLAST search against the KEGG database, and the top-ranked homologous sequences were used for multiple sequence alignment and preparation of the phylogenetic tree by Muscle (https://www.ebi.ac.uk/jdispatcher/msa/muscle?stype=protein) [46]. The scale bar is shown above the tree. The values to the right of the protein names represent branch lengths. Groups 1–3 are defined based on the visual pattern of the tree.

Group	Protein name	Trp74	Asn75	Asn131	Glu229	Trp292	Glu293	Arg303	Glu305	Trp308	Gln312	Asp336	Glu415	Lys419	Trp429	Glu430	Trp446
Group 1	Chy400_4166	**W**	**N**	**N**	**E**	**W**	**E**	**R**	**E**	**W**	**Q**	**D**	**E**	**K**	**W**	**E**	**W**
	Cagg_0090	**W**	**N**	**N**	**E**	**W**	**E**	**R**	**E**	**W**	**Q**	**D**	**E**	**K**	**W**	**E**	**W**
	ANT_26400	**W**	**N**	**N**	**E**	**W**	**E**	**R**	**E**	**W**	**N**	**D**	**E**	**K**	**W**	**E**	**W**
	CLDAP_22410	**W**	S	D	**E**	**W**	**E**	I	A	**Y**	G	**D**	**E**	**K**	**W**	**E**	**W**
	NCTC10138_01618	**W**	**N**	S	G	G	S	**K**	**E**	**Y**	**N**	**D**	**E**	**K**	**W**	**E**	A
	BN85304570	**W**	**N**	D	N	G	N	T	A	**Y**	**N**	**D**	**E**	**K**	**W**	Q	S
	BK011_09400	**W**	**N**	G	A	**W**	V	Q	**D**	**W**	S	**D**	**E**	**K**	**F**	T	G
	G4Z02_04695	**W**	**N**	G	T	G	F	N	**D**	**W**	L	**D**	**E**	**K**	**F**	W	G
Group 2	Ferpe_0815	**W**	**N**	P	I	G	**D**	**R**	**D**	**W**	F	**D**	**E**	**K**	**W**	T	G
	IB67_02045	**W**	**N**	P	V	G	**D**	**R**	S	**W**	F	**D**	**E**	**K**	**W**	T	G
	Theba_2482	**W**	**N**	P	V	G	**D**	**R**	S	**W**	S	**D**	**E**	**K**	**W**	T	G
	JRV97_09585	**W**	**N**	P	V	G	**E**	**R**	S	**W**	**N**	**D**	**E**	**K**	**W**	T	G
	GYMC10_3166	**W**	**N**	P	V	G	**D**	**R**	**E**	**W**	D	**D**	**E**	**K**	**W**	**E**	G
	HM131_05720	**W**	S	T	N	T	**E**	M	G	**W**	G	**D**	**E**	**K**	**W**	**E**	G
	PQ477_07890	**W**	G	A	F	E	**E**	**K**	**D**	**W**	G	**D**	**E**	**K**	**W**	**E**	A
	B4U37_18785	**W**	A	S	F	G	**E**	N	**D**	**W**	**N**	**D**	**E**	**K**	**W**	**E**	A
	ABE65_018500	**W**	S	P	F	E	**E**	**K**	**D**	**W**	A	**D**	**E**	**K**	**W**	**E**	A
	NRE15_04935	**W**	**N**	**N**	G	G	L	N	**D**	**W**	T	**D**	**D**	**K**	**F**	**E**	G
	OTR81_04320	**W**	**N**	**N**	Q	G	A	Y	N	**Y**	D	**D**	**D**	**K**	**F**	**E**	**W**
Group 3	L1F29_16565	**W**	M	F	G	-	W	Q	**D**	**W**	G	**D**	**D**	**K**	S	V	-
	L1F29_27145	**W**	M	F	G	-	W	Q	**D**	**W**	G	**D**	**D**	**K**	D	V	-
	IJ21_25970	**W**	M	F	K	-	S	Q	**D**	**W**	G	**D**	**D**	**K**	N	I	-
	EJC50_24715	**W**	G	F	A	-	S	V	**D**	**W**	W	**D**	**E**	**K**	S	V	-
	bsdcttw_05710	**W**	G	F	N	A	W	H	**D**	**W**	Y	**D**	T	S	**W**	A	-
	CDO33_02425	**W**	G	F	I	A	W	H	**D**	**W**	H	**D**	S	P	**W**	M	-
	UB51_23540	**W**	E	E	W	A	W	G	**D**	**W**	S	**D**	**D**	**K**	**W**	A	-

Recognition of unit	Trp74	Asn75	Asn131	Glu229	Trp292	Glu293	Arg303	Glu305	Trp308	Gln312	Asp336	Glu415	Lys419	Trp429	Glu430	Trp446
A																
B															B	B
C				C												
D			D											**D**	D	
E												**E**				
F	**F**															
G	**G**	G							**G**		**G**		**G**			
H																
I		I							**I**							
J							J	J	J	J						
K and beyond					M	L										

Figure S4. Conservation of substrate recognition residues.

Conserved residues within Groups 1–3 are indicated by bold red font and a light red highlight. In the case of chemically conserved residues, the residues are shown in bold black letters. The other residues conserved with Chy400_4166 are indicated by the same font and a light green highlight. In the case of chemically conserved residues, they are shown in bold black letters and highlighted in light blue. Units and their recognizing residues are consistently highlighted in light red. Unit J is not highlighted because its interaction with Trp308, which is substituted by Tyr in some proteins, is a hydrogen bond.

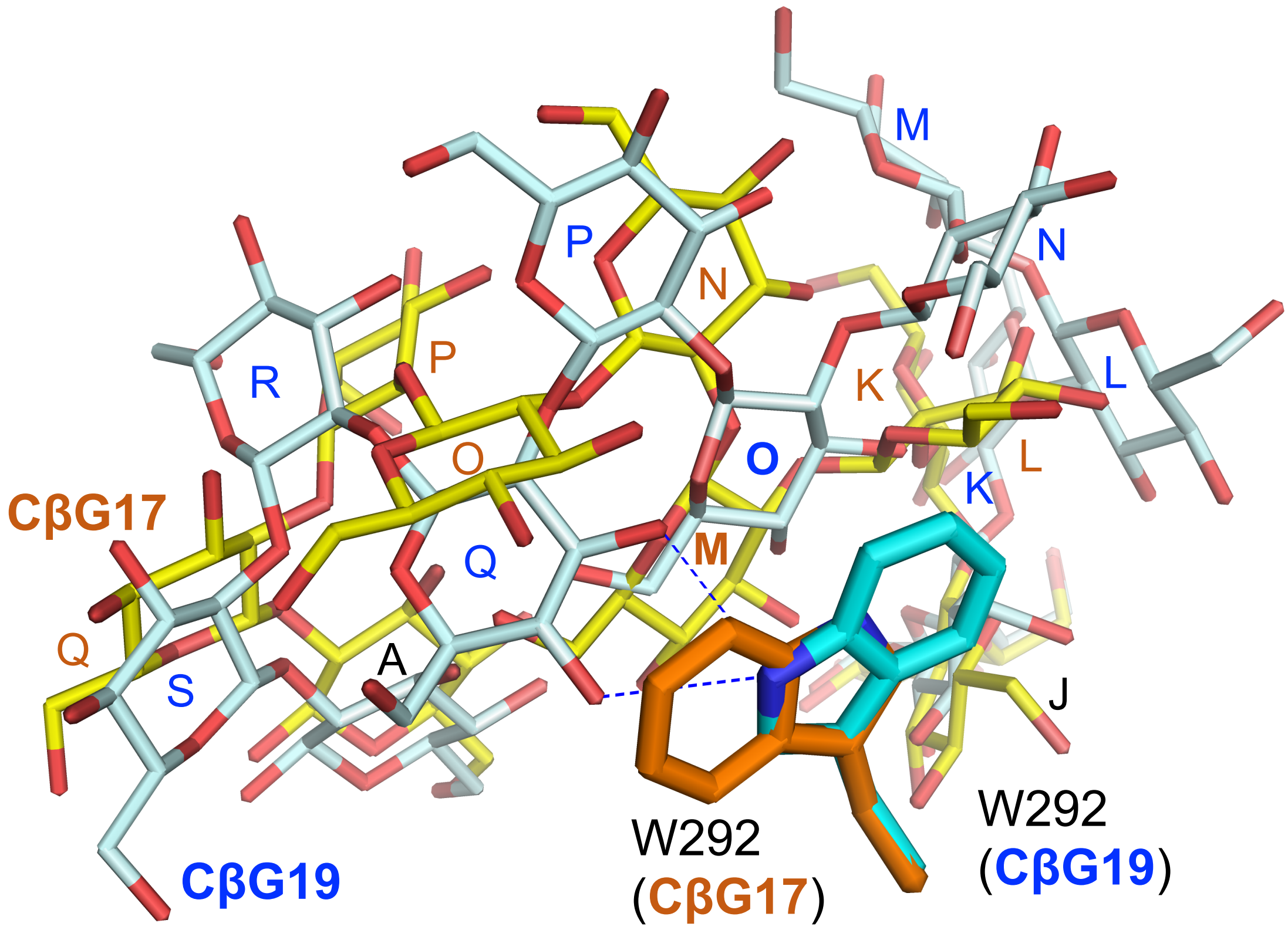

Figure S5. Comparison of recognition of CβG17 and CβG19 by W292.

CβG17 and CβG19 are shown as yellow and light blue thin sticks, respectively. The defined unit names in CβG17 and CβG19 are labelled in black and blue letters, respectively. W292 residues in CβG17 and CβG19 complexes are shown in orange and cyan thick sticks, respectively. Hydrogen bonds between CβG19 and W292 are indicated by dashed blue lines. PyMOL was used for structural representation.

Supplementary note

Supplementary note 1, “S” is an abbreviation of “sophoro”. Sophorooligosaccharide is an alternative name of β-1,2-glucooligosaccharide. The reason why “S” is used for abbreviation is to distinguish linkage positions from other β-linked glucooligosaccharides and enzymes acting on β-linked glucose polymers clearly.
